# Age-Related Changes in Virtual Pivot Point Position and Variability During Pediatric Gait Development

**DOI:** 10.3390/children13030363

**Published:** 2026-03-03

**Authors:** Lucas Schreff, Katharina Nirmaier, Christian Blank, Rainer Abel, Roy Müller

**Affiliations:** 1 Department of Orthopedic Surgery, Klinikum Bayreuth GmbH, 95445 Bayreuth, Germany; 2 Social Pediatrics Centre, LA-Regio Kliniken—Kinderklinik St. Marien, 84036 Landshut, Germany; 3 Universitätsklinikum Erlangen, Friedrich-Alexander-Universität Erlangen-Nürnberg, 91054 Erlangen, Germany; 4 Bayreuth Center of Sport Science, University of Bayreuth, 95447 Bayreuth, Germany

**Keywords:** virtual pivot point (VPP), toddlers, pediatric walking, gait development, ground reaction forces (GRF), postural control, gait variability, markerless motion capture

## Abstract

**Highlights:**

**What are the main findings?**

- Even in pediatric walking, ground reaction forces focus towards a virtual pivot point.
- VPP position and variability change with age, reflecting maturation of gait control.

**What are the implications of the main findings?**

- Age-related VPP changes indicate developmental shifts in trunk stabilization strategies.
- Understanding VPP development may support early identification of atypical gait patterns.

**Abstract:**

**Background/Objectives**: During adult walking, ground reaction forces (GRFs) consistently intersect near a point above the center of mass (CoM), termed the virtual pivot point (VPP). The VPP is hypothesized to contribute to upper body stabilization. However, little is known about its presence and developmental trajectory during early childhood. This study investigated age-related differences in VPP position, variability, and GRF focusing during walking in typically developing children. **Methods**: Kinematic and kinetic data were collected from 29 children across three age groups: Group I (aged 1 year), Group II (aged 2–3 years), and Group III (aged 10–15 years) using markerless motion capture and force plates. VPP position relative to the CoM, its variability and GRF focusing (*R*^2^) were analyzed in sagittal plane during single support phases. **Results**: Across all age groups, GRFs were strongly focused toward a VPP (*R*^2^ > 0.95), with no significant age-related differences in GRF focusing. In contrast, significant age-related differences emerged in VPP position and variability. The normalized vertical VPP position increased progressively from Group I (7.58 cm) to Group III (14.79 cm). Notably, in several toddlers, the VPP was located at or below the CoM, contrasting with the consistent above-CoM position observed in adolescents. **Conclusions**: These findings demonstrate that while GRF focusing behavior is present in toddlers who can walk independently, VPP characteristics undergo substantial developmental changes. The shifting VPP position and the decrease in variability in toddlers likely reflect progressive changes in gait mechanics and trunk stabilization strategies during childhood.

## 1. Introduction

In bipedal locomotion, the ability to maintain the upper body and consequently the entire body in an upright posture is a key competence, because humans have a much smaller base of support when walking than quadrupeds [1,2]. Consequently, infants usually develop the motor skills necessary for standing and walking on two legs after they have first learnt to crawl on their hands and knees [3,4]. During bipedal walking, the feet must be positioned so that ground reaction forces (GRFs) can be used to control and stabilize the center of mass (CoM) motion and whole body angular momentum [5,6]. Experimental studies [1,7,8,9] of human adult walking have demonstrated that GRFs during the single support phase intersect in the sagittal plane near a point above the CoM, referred to as the virtual pivot point (VPP). It is hypothesized that the VPP contributes to the stabilization of human gait [1,10].

In experimental studies on adult gait, the characteristic focusing of GRFs toward the VPP has been observed across a wide range of locomotor conditions, including walking at different speeds [1,8,11], hip-flexed walking [9], descent over both visible and camouflaged ground perturbations [10], and even backward walking [7]. However, neither the degree to which the GRFs focus on the VPP nor the position of the VPP relative to the CoM are fixed properties. Instead, they can vary depending on age [12], the specific locomotor task [7], as well as individual neuromuscular or biomechanical constraints [13,14].

Age-related differences in VPP characteristics have been previously described, with younger participants exhibiting significantly greater GRF focusing toward the VPP compared to older individuals, indicating that the VPP parameters change across the lifespan [12]. Against this background, it remains unclear how pronounced these age-related differences are in the early stages of independent walking. The GRFs of toddlers deviate from those of adults [15]. Even when normalized, all three components of GRFs show age-related changes up to approximately five years of age, after which an adult-like pattern emerges [16]. These developmental differences are reflected in the magnitude and timing of the GRFs in relation to body weight [17,18]. The characteristic second peak of vertical GRFs observed in adults walking is almost completely absent in children who are just learning to walk independently and only begins to increase with age [15,19,20].

Given these developmental differences in GRF characteristics, it remains unclear whether toddlers generate a VPP comparable to that observed in adults and, if so, how its location and stability evolve with age. A detailed understanding of age-related changes in VPP characteristics may also be clinically relevant, as trunk stabilization and whole-body control are frequently altered in children with developmental motor disorders (e.g., [21,22]). Establishing normative developmental trajectories of VPP position and variability may therefore contribute to improved interpretation of pediatric gait analyses and support early identification of atypical motor development.

To address these questions, we analyzed GRFs and kinematic data from three age groups of typically developing children, ranging from toddlers who could walk independently to adolescents, using synchronized markerless motion capture and force plate measurements. By computing the intersection point of GRFs in the sagittal plane across multiple steps, we assessed the presence, position, variance, and degree of GRF focusing toward a VPP. We hypothesized that (1) the VPP position, its variability, and the extent of GRF focusing would differ substantially between the youngest children and the adolescent reference group, and that (2) these differences would be reduced in the intermediate age group, reflecting the progressive maturation of gait mechanics and neuromuscular control.

## 2. Materials and Methods

### 2.1. Participants

**Figure 1:**
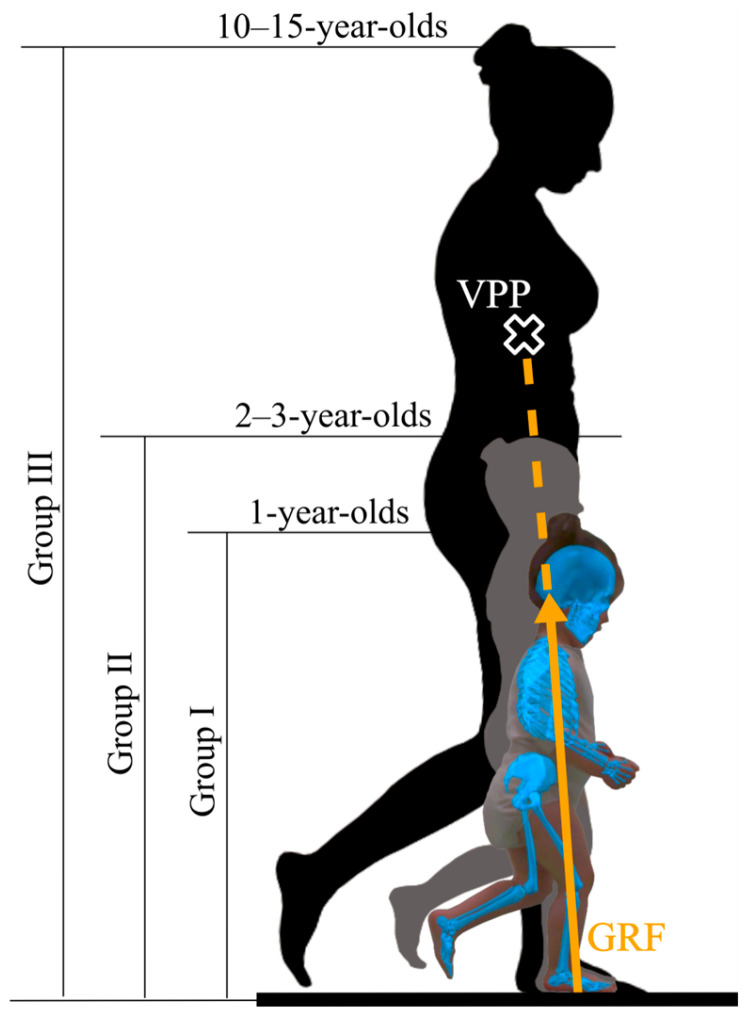
Representative silhouettes in the sagittal plane during single-support phase of gait for the three age groups. The light blue skeletal model (silhouette of Group I) shows the output of the markerless 3D motion capture system. The yellow ground reaction force (GRF) vector (silhouette of Group III) points to the virtual pivot point (VPP, marked as a white-framed X).

In this study a dataset from the normative database of the Motion Analysis Laboratory at LA-Regio Kliniken – Kinderklinik St. Marien (Landshut, Germany) was used. From this database, we retrospectively analyzed 29 datasets of children between 1 and 15 years of age and divided them into three groups (Table 1, Figure 1). Group I consisted of 1-year-old children who had learned to walk independently within the last 12 months. Group II included children aged 2–3 years who had been walking independently for more than 12 months. Group III comprised adolescents aged 10–15 years, serving as a reference group with adult-like GRF patterns. Participant’s characteristics can be seen in Table 1.

Inclusion criteria were: (1) age-appropriate normal development (anthropometric and pediatric); (2) no history of orthopedic or neurological disorders; (3) no gait abnormalities. Additionally, each child underwent a clinical examination to assess the functional status of the musculoskeletal system. The clinical examination was performed collaboratively by two experienced physiotherapists who have completed several advanced training courses in gait analysis. Both examiners jointly assessed every participant to ensure consistent evaluation procedures. All data was collected in accordance with the ethical principles of the Declaration of Helsinki. Written informed consent was obtained from the parents or legal guardians of all participants.

### 2.2. Measurements

Participants walked barefoot at their self-selected comfortable speed while wearing tight-fitting clothing to ensure accurate motion capture. Kinematic data were collected markerless using the Qualisys Track Manager (11 Miqus cameras (100 Hz), Qualisys AB, Gothenburg, Sweden) in combination with Theia3D (Theia Markerless, Inc., Kingston, ON, Canada). Theia3D used deep learning–based computer vision algorithms to automatically identify anatomical landmarks (currently 124) from video recordings. These landmarks were reconstructed in 3D and subsequently transferred into Visual3D (C-Motion, Inc., Germantown, MD, USA). In Visual3D, the landmarks were assigned to body segments, segment coordinate systems were created, and joint centers are calculated to generate a biomechanical model. Prior to data collection, the cameras were calibrated using a calibration wand according to the procedure recommended by Qualisys AB (calibration time: 60 s). It should be noted that markerless motion capture is not yet the gold standard for analyzing human movements (e.g., [23,24]). Compared to marker-based systems, a systematic review reports the best validity and reliability of kinematic data in the sagittal plane and notes a lack of valid and accurate outcomes in the frontal and transversal planes [25]. In the present study, kinematic data was used solely for calculating the CoM in sagittal plane. A previous validation study has demonstrated good agreement between markerless and marker-based systems for CoM trajectory estimation [26].

Ground reaction forces (1000 Hz) were collected synchronously with kinematic data using two floor-integrated force plates (Bertec Corporation, Columbus, OH, USA) and subsequently transferred to the Visual3D model for kinetic analysis. The force plates were zeroed prior to each of the participants’ trials.

### 2.3. Data Processing

The raw kinetic data were filtered using a low-pass Butterworth filter with a cut-off frequency of 25 Hz. The raw kinematic data were filtered using a Generalized Cross-Validatory Spline filter [27] with a cut-off frequency of 12 Hz. These filter settings are similar to those used in previous markerless motion capture studies [23,28,29].

For the group of children aged 13–15 years, two trials per participant were analyzed in which both force plates were each contacted by a single foot without crossing plate boundaries. For the groups of children aged 1 year and 2–3 years, one representative trial per participant was analyzed in which the child walked straight across the force plates and performed three to four consecutive foot contacts (depending on step length). To ensure accurate center of pressure (CoP) data in cases of potential cross-plate strikes, we used Equation (1) [30],

(1) $$a=a_{1}\times \left(\frac{{Fz}_{1}}{Fz}\right)+a_{2}\times \left(\frac{{Fz}_{2}}{Fz}\right)$$

where *a* denotes the global location of the CoP vector, *a*_1*/*2_ the CoP location measured by force plate 1 or 2, *Fz*_1*/*2_ the vertical force measured by force plate 1 or 2, and *Fz* the combined vertical force measured by both force plates.

The whole-body center of mass (CoM) was calculated in Visual3D as the weighted sum of individual segment centers of mass. Segment masses and the relative positions of segmental CoM locations were determined using established anthropometric scaling parameters [31] based on participants’ body mass and segment lengths derived from the kinematic model. The same relative segment mass parameters were applied across all age groups.

To determine the VPP we adopted the calculation script of Vielemeyer et al. [11]. All GRFs from the single support phase were transformed into a CoM-centered coordinate system, with each vector originating from the CoP. The VPP was then identified as the point at which the sum of the squares of the perpendicular distances between the GRF vectors and this point was minimized. This optimization procedure determines the position of the VPP relative to the CoM for each step. The focusing of GRFs toward the VPP was quantified using the coefficient of determination (*R*^2^, Equation (2)), adapted from Herr and Popovic [32] and Vielemeyer et al. [10]:

(2) $$R^{2}=1-\frac{\sum_{i}^{N}{\left(\theta^{i}-\theta_{VPP}^{i}\right)}^{2}}{\sum_{i}^{N}{\left(\theta^{i}-\bar{\theta }\right)}^{2}}$$

where $\theta_{\mathrm{VPP}}^{i}$ represents the theoretical angle of a GRF pointing directly from the CoP to the VPP at each time point i, and $\theta^{i}$ denotes the corresponding angle of the measured GRF with respect to the ground. $\overset{‾}{\theta }$ is the mean of all measured angles $\theta^{i}$. An *R*^2^ value of 1 indicates perfect agreement between theoretical and measured GRF directions, reflecting ideal focusing of all GRFs onto the VPP. Reduced or even negative *R*^2^ values represent decreased focusing and therefore weaker alignment of the GRFs toward a single intersection point. In previous studies investigating level walking in healthy participants, *R*^2^ values of ≥0.95 have been reported. Based on the work of Herr and Popovic [32], subsequent VPP studies [7,13] applied a threshold of *R*^2^ > 0.6 to define the presence of a meaningful GRF intersection point. Other studies have used a threshold of *R*^2^ > 0.8 [12,14].

### 2.4. Statistical Analysis

Statistical analyses were performed using SPSS Statistics (version 20.0, IBM Corp., Chicago, IL, USA). Homogeneity of variances was assessed using Levene’s test. Differences between age groups were evaluated using one-way ANOVA (*p* < 0.05) with post hoc analysis (Tamhane). Measures of effect size were provided as eta squared (*η*^2^). Values of 0.01 were considered as weak effects, 0.06 as moderate effects, and 0.14 as strong effects [33]. The parameters included in the statistical analyses were walking speed (mean of horizontal CoM speed: *v*), relative duration of the single support phase (*SSP*), GRF focusing toward the VPP (*R*^2^), and the position of the VPP relative to the CoM in the sagittal plane. All parameters were calculated for each individual step, defined as the interval from heel strike to the subsequent heel strike of the same foot. For between-group comparisons, the vertical and horizontal VPP positions were normalized to body height (*VPP_h_norm_; VPP_v_norm_*). In addition, 95% confidence ellipses were calculated to characterize the spatial variability of the VPP within each group. To specifically assess gait consistency during early development, inter-step variability of *VPP_v_norm_* was analyzed for Group I and Group II. The standard deviation of *VPP_v_norm_* across consecutive steps within the analyzed trial was therefore calculated for all children in these groups. These values were then correlated with the age (in months) of all Group I and II participants. The strength of the Pearson correlation coefficient was interpreted according to Cohen [33]: r ≈ 0.10 was considered weak, 0.10 < r < 0.30 was considered moderate, and r ≥ 0.50 was considered strong.

## 3. Results

Across all age groups, ground reaction forces (GRFs) were strongly focused toward an intersection point during single support, with group mean *R*^2^ values exceeding 0.95 in all groups (Table 2). However, the one-way ANOVA revealed significant main effects of group for gait speed (strong effect size, Table 2), duration of the single support phase (strong effect size) as well as for normalized horizontal and vertical VPP positions (each moderate effect size).

### 3.1. Age-Dependent Mean Differences

Gait speed increased progressively with age, with Group I walking at 0.86 m/s and Group II at 0.90 m/s, both significantly slower than Group III (1.20 m/s; Table 2). No significant difference in gait speed was observed between Groups I and II. The relative duration of the single support phase also increased progressively with age, accounting for 47.91% of the gait cycle in Group I and 50.14% in Group II, both of which were significantly smaller than in Group III (56.38%). No significant difference in single support duration was observed between Groups I and II.

The normalized horizontal VPP position differed significantly between groups, with Group II exhibiting a smaller horizontal displacement relative to the CoM (−0.15 cm) compared to Group III (−0.82 cm), while Group I (−0.28 cm) did not differ significantly from either group. Significant age-related differences were also found for the normalized vertical VPP position. Group I showed a markedly lower vertical VPP position (7.58 cm), corresponding to approximately half the height observed in Group III (14.79 cm; Table 2). Group II exhibited an intermediate vertical VPP position (11.43 cm), which lay between Groups I and III but did not differ significantly from either group.

### 3.2. Age-Dependent Variability

**Figure 2:**
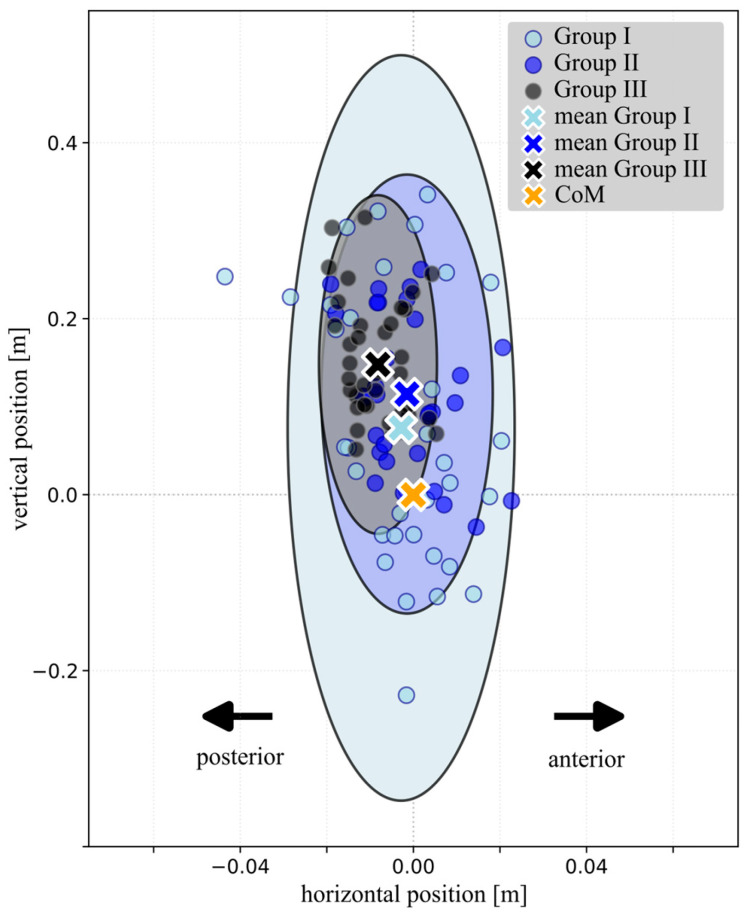
Distribution of virtual pivot point (VPP) positions (normalized to body height) relative to the center of mass (CoM) in the sagittal plane for the three age groups. Individual VPP locations and group-specific 95% confidence ellipses are shown. The extent of the ellipses reflects the variability of VPP position within each group. Note: Axes are scaled 5:1 (x:y) for visualization purposes.

Levene’s test revealed significant variance heterogeneity for all parameters listed in Table 2 (all *p* < 0.05), whereas the relative single support phase showed no significant deviation from variance homogeneity (*p* = 0.06). This finding is reflected in Figure 2, which illustrates group-specific 95% confidence ellipses of VPP positions. Group I exhibited a markedly larger ellipse compared to Groups II and III, indicating substantially greater variability in both vertical and horizontal VPP location. This increased variability is also reflected in the increased standard deviations reported for Group I, which progressively decreased in Group II and III (Table 2). To further quantify step-to-step consistency during early gait development, inter-step variability of *VPP_v_norm_* was analyzed for Groups I and II. *VPP_v_norm_* variability showed a strong negative correlation with age in months (*r* = −0.586, *p* < 0.05), indicating a progressive reduction in variability with increasing age.

### 3.3. VPP Location Relative to the CoM

**Figure 3:**
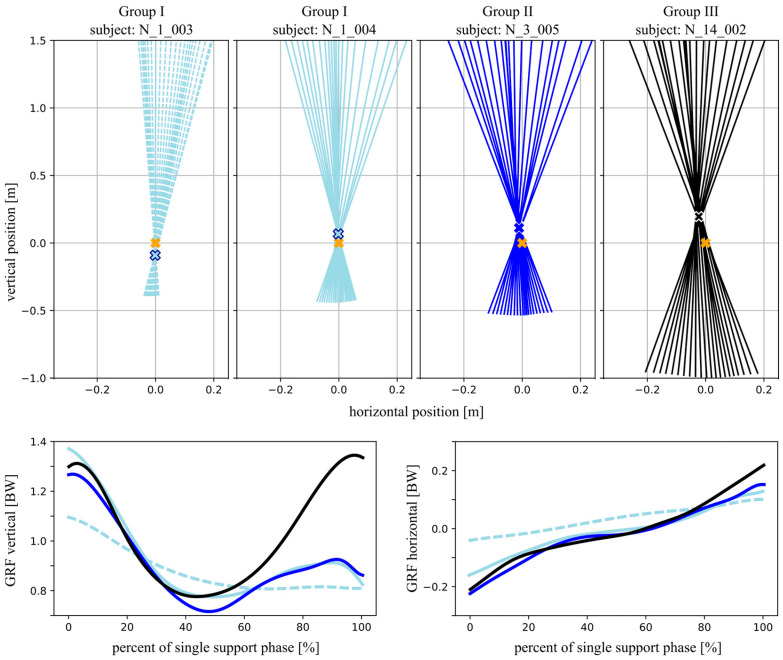
Representative virtual pivot point (VPP) plots from individual participants of Groups I (dashed and solid light blue lines), II (dark blue line), and III (black line). The yellow cross indicates the position of the center of mass (CoM). Colored crosses (light blue, blue, and black) denote the corresponding VPP locations. For Group I, two examples are shown, illustrating cases with the VPP located below and above the CoM, respectively. The bottom panels show the corresponding (BW body weight) normalized vertical and horizontal ground reaction forces (GRFs) during the single support phase.

Figure 2 shows that the VPP was located at the level of or below the CoM in several Group I participants, while in other Group I children it was positioned above the CoM. Occasional VPP locations at or below the CoM were also observed in Group II, whereas VPPs in Group III were located above the CoM. Figure 3 shows representative VPP plots from individual participants of the three groups. Two examples are presented for Group I, illustrating one case with a VPP located below the CoM and one with a VPP located above the CoM. The corresponding normalized GRF profiles during the single support phase are also shown. In the vertical GRF component, children from Groups I and II exhibited a reduced or absent second peak compared to the characteristic double-peaked pattern observed in the adolescent Group III. Differences in the horizontal GRF components were smaller. The child from Group I with a VPP located below the CoM showed reduced horizontal GRF magnitudes compared to the other examples.

## 4. Discussion

Although the virtual pivot point (VPP) has been extensively investigated in adults, little is known about its development during early childhood walking. In particular, it remains unclear whether toddlers exhibit a stable VPP comparable to mature gait and how its position and variability change with age. Against this background, this study investigated age-related differences in the VPP during walking in terms of its position, variability, and the degree of GRF focusing (*R*^2^). Our results largely confirm our hypotheses, demonstrating that both the position and the variability of the VPP are age dependent and reflect developmental changes in gait. In contrast, no significant age-related differences were observed in GRF focusing (*R*^2^).

In the present study, *R*^2^ values (≥0.95, Table 2) were consistently larger across all age groups, indicating a strong focusing of GRFs toward a virtual intersection point. This finding suggests that even children who have only recently acquired independent walking already exhibit a well-defined GRF focusing behavior. Age-related differences in *R*^2^ have been reported previously by Firouzi et al. [12], who observed significantly smaller *R*^2^ values in older adults (mean age: 62.7 years) compared with a young adult control group (mean age: 27.6 years). In contrast to our results, these differences reached statistical significance. This discrepancy is likely attributable to differences in statistical approach and experimental design. Notably, despite the reported age-related differences in GRF focusing, Firouzi et al. [12] did not observe significant group differences in the sagittal-plane VPP position.

However, our results showed significant age-related differences observed in the normalized vertical VPP position and its variability (Table 2). In several children from Group I and in some children from Group II, the VPP was located at the level of or even below the CoM (Figure 2). This altered vertical VPP position and the increased variability observed in younger children may be related to developmental characteristics of early gait. It is known that toddlers frequently vary their walking speed [34] which could affect the position and variability of the VPP. To date, a VPP located below the CoM has been reported for running in grown-up participants, where vertical GRFs typically exhibit a single peak rather than the characteristic double-peak pattern observed during adult walking [35,36]. As illustrated in Figure 3, the second peak of the vertical GRF was weakly developed and variable in the younger children of our cohort. To what extent a reduced or absent second vertical GRF peak directly influences the vertical VPP position remains an open question and should be addressed in future studies.

A VPP located above the CoM is thought to generate a restoring moment relative to the whole-body CoM that passively stabilizes the upper body during walking ([1]; see Figure 1 in [9]). In contrast, when the VPP is located below the CoM, the resulting ground reaction force vector generates a moment relative to the CoM that acts in the opposite rotational direction to the restoring moment observed when the VPP is above the CoM and does not contribute to passive trunk stabilization. In toddlers, increased trunk oscillations are commonly observed [37,38], suggesting that upper-body stabilization relies more strongly on active control strategies rather than passive mechanical mechanisms. One such strategy may be the characteristic “high-guard” arm posture, which enables children to modulate moment and explore balance control during early stages of gait development [39]. With increasing age and walking experience, trunk oscillations gradually decrease and the arms are lowered, changes that are typically accompanied by increased walking speeds and improved gait efficiency [37,39,40].

Regarding the horizontal VPP position, the normalized horizontal location differed significantly between Groups II and III, whereas no significant difference was observed between Groups I and III. This pattern does not indicate a linear age-related trend in horizontal VPP displacement. However, the interpretation of these findings is complicated by the normalization of horizontal VPP position to body height. While normalization improves comparability across individuals of different sizes, its influence on the horizontal direction remains unclear and warrants further investigation [13].

Further limitations of this study should be acknowledged. First, the relatively small sample size limits statistical power. However, effect sizes (*η*^2^) for the investigated parameters were moderate to strong, suggesting meaningful age-related differences. Future studies with larger samples and an a priori power analysis should investigate potential determinants of VPP position, such as walking speed, percentage single support phase, kinematics, kinetics, and sex. In addition, substantial inter-individual differences in developmental stage, especially among toddlers, limit the ability to form homogeneous groups. Secondly, to the best of our knowledge, this study is the first VPP investigation to use markerless motion capture. However, the VPP values determined for the adolescent age group (Group III) are comparable to previously published marker-based data from a healthy control group (mean age: 15.5 years; mean height: 158 cm; *VPP_v_norm_*: 14.0 cm; *VPP_h_norm_*: 0.1 cm, [13]). Furthermore, the calculation of the CoM introduces additional uncertainty in the estimation of the VPP position relative to the CoM. The choice of anthropometric model influences the CoM movement during walking [41]. In this study, segment mass distributions were derived from adult anthropometric data, whereas a previous study has shown that body segment mass distribution in children is age-dependent and differs from that of adults [42]. We hypothesize that due to the more cranially distributed mass in toddlers, the VPP would shift caudally relative to the CoM in Groups I and II when age-appropriate mass distributions are considered.

In summary, this study shows that the position and variability of the VPP are age-dependent and reflect developmental changes, whereas the degree of GRF focusing remains largely stable across all age groups. These findings suggest that the maturation of GRFs and VPPs throughout childhood could offer insight into how the trunk stabilization strategy develops during walking. Future longitudinal research is required to characterize the developmental trajectories of VPP parameters at an individual level and establish whether they could serve as clinically meaningful indicators of motor development. A deeper understanding of gait maturation could support the identification and interpretation of pathological gait patterns in early childhood [43].

## Figures and Tables

**Table 1:** Participant’s characteristics.

	Group I 1-Year-Olds	Group II 2–3-Year-Olds	Group III 10–15-Year-Olds
Sex [w/m]	5/5	5/4	5/5
Age [years]	1.5 ± 0.3	3.0 ± 0.6	12.3 ± 2.1
Height [cm]	81.2 ± 4.0	94.4 ± 6.3	159.7 ± 12.4
Body mass [kg]	11.2 ± 1.2	14.5 ± 1.4	46.3 ± 12.0

**Table 2:** Group means (±SD) for gait speed (*v*), percentage single support phase (*SSP*), GRF focusing coefficient (*R*^2^), and normalized vertical and horizontal VPP positions relative to the CoM (*VPP_h_norm_*, *VPP_h_norm_*) for the three age groups. Measures of effect size were provided as eta squared (*η*^2^). ^A^ denotes significant differences between Group I and Group III; ^B^ denotes significant differences between Group II and Group III.

	Group I 1-Year-Olds	Group II 2–3-Year-Olds	Group III 10–15-Year-Olds	*η* ^2^	
*n*	36	31	40		
*v* [m/s]	0.86 ± 0.21	0.90 ± 0.18	1.20 ± 0.12	0.46	^A,B^
*SSP* [%]	47.91 ± 6.02	50.14 ± 6.34	56.38 ± 4.65	0.31	^A,B^
*R* ^2^	0.95 ± 0.10	0.98 ± 0.02	0.98 ± 0.02	0.07	
*VPP_h_norm_* [cm]	−0.28 ± 1.13	−0.15 ± 1.01	−0.82 ± 0.66	0.08	^B^
*VPP_v_norm_* [cm]	7.58 ± 14.96	11.43 ± 8.73	14.79 ± 6.89	0.08	^A^

## Data Availability

The raw data supporting the conclusions of this article will be made available by the authors on request. The data are not publicly available due to privacy reasons.

## Abbreviations

The following abbreviations are used in this manuscript:

BW	Body Weight
CoM	Center of Mass
CoP	Center of Pressure
GRF/GRFs	Ground Reaction Force(s)
R^2^	Coefficient of Determination (focusing of GRFs)
SD	Standard Deviation
SSP	relative duration of the Single Support Phase
v	gait speed
VPP	Virtual Pivot Point
VPP_h_norm_	horizontal VPP position, normalized to body height
VPP_v_norm_	vertical VPP position, normalized to body height
η^2^	eta squared (effect size)
